# Inflammatory Proteins HMGA2 and PRTN3 as Drivers of Vulvar Squamous Cell Carcinoma Progression

**DOI:** 10.3390/cancers13010027

**Published:** 2020-12-23

**Authors:** Agnieszka Fatalska, Natalia Rusetska, Elwira Bakuła-Zalewska, Artur Kowalik, Sebastian Zięba, Agnieszka Wroblewska, Kamil Zalewski, Krzysztof Goryca, Dominik Domański, Magdalena Kowalewska

**Affiliations:** 1 Mass Spectrometry Laboratory, Institute of Biochemistry and Biophysics-Polish Academy of Sciences, 02-106 Warsaw, Poland; fataga@wp.pl (A.F.); dom.domanski@ibb.waw.pl (D.D.); 2 Department of Molecular and Translational Oncology, Maria Sklodowska-Curie National Research Institute of Oncology, 02-781 Warsaw, Poland; natarusetska@gmail.com (N.R.); zalewski81@gmail.com (K.Z.); 3 Department of Pathology, Maria Sklodowska-Curie National Research Institute of Oncology, 02-781 Warsaw, Poland; elwirabz@onet.eu; 4 Department of Molecular Diagnostics, Holycross Cancer Center, 25-734 Kielce, Poland; arturko@onkol.kielce.pl (A.K.); s.zieba@o2.pl (S.Z.); 5 Division of Medical Biology, Institute of Biology, Jan Kochanowski University, 25-369 Kielce, Poland; 6 Department of Gynecology, Holy Family Hospital, 02-544 Warsaw, Poland; agnwr@op.pl; 7 Department of Gynecologic Oncology, Holycross Cancer Center, 25-734 Kielce, Poland; 8 Chair and Department of Obstetrics, Gynecology and Oncology, 2nd Faculty of Medicine, Warsaw Medical University, 03-242 Warsaw, Poland; 9 Department of Genetics, Maria Sklodowska-Curie National Research Institute of Oncology, 02-781 Warsaw, Poland; kgoryca@gmail.com; 10 Genomics Core Facility, Centre of New Technologies, University of Warsaw, 02-097 Warsaw, Poland; 11 Department of Immunology, Biochemistry and Nutrition, Centre for Preclinical Research and Technologies, Medical University of Warsaw, 02-097 Warsaw, Poland

**Keywords:** HMGA2, iTRAQ, microbiome, proteomics, PRTN3, vulvar carcinoma

## Abstract

**Simple Summary:**

Our study aimed to advance the understanding of vulvar squamous cell carcinoma (VSCC) biology by recognizing biological pathways that drive the progression of this disease. We applied the experimental path from global proteomic analysis of vulvar tumors to the targeted and quantitative assessment of specific proteins both in the tumors and blood of VSCC patients. The proteomic analysis has advanced the knowledge on VSCC biology by pointing at inflammation as a driver of progression and by providing grounds for the hypothesis of vulvovaginal microflora disturbances as a trigger for the inflammatory response. The study results indicate prognostic protein markers and potential therapeutic targets for improved and personalized management of VSCC.

**Abstract:**

Current knowledge on the biology of squamous cell vulvar carcinoma (VSCC) is limited. We aimed to identify protein markers of VSCC tumors that would permit to stratify patients by progression risk. Early-stage tumors from patients who progressed (progVSCC) and from those who were disease-free (d-fVSCC) during follow-up, along with normal vulvar tissues were examined by mass spectrometry-based proteomics. Differentially expressed proteins (DEPs) were then verified in solid tissues and blood samples of patients with VSCC tumors and vulvar premalignant lesions. In progVSCC vs. d-fVSCC tumors, the immune response was the most over-represented Gene Ontology category for the identified DEPs. Pathway profiling suggested bacterial infections to be linked to aggressive VSCC phenotypes. High Mobility Group AT-Hook 2 (HMGA2) and Proteinase 3 (PRTN3) were revealed as proteins predicting VSCC progression. HMGA2 and PRTN3 abundances are associated with an aggressive phenotype, and hold promise as markers for VSCC patient stratification. It appears that vulvovaginal microflora disturbances trigger an inflammatory response contributing to cancer progression, suggesting that bacterial rather than viral infection status should be considered in the development of targeted therapies in VSCC.

## 1. Introduction

Vulvar carcinoma is a rare genital malignancy contributing to the estimated 44,235 new cases and 15,222 cancer-related deaths in 2018 [1]. Surgical management is at the cornerstone of treatment for most vulvar cancers, while radiotherapy is added in advanced cases. Vulvar carcinoma spreads primarily by local expansion and the lymphatic system. The status of the regional lymph nodes (LN) is the most important prognostic factor for vulvar carcinoma patients and therefore it is included in the International Federation of Gynecology and Obstetrics and American Joint Committee on Cancer TNM staging systems for vulva. The five-year overall survival rates are approximately 85.5% and 50.6% in patients without and with LN involvement, respectively [2]. However, the LN status can only be determined by histological examination of the resected LNs, i.e., lymphadenectomy or sentinel LN biopsy, which are performed in patients eligible for surgery [3]. Thus, these invasive and often highly morbid surgeries cannot be avoided as their benefits outweigh the risk of not detecting the cancer spread in the regional LNs and thus the risk of relapse. Newer strategies, combining surgery, radio- and chemotherapy, and tailoring the treatment to the extent of clinical and pathologic disease, are used in advanced and metastatic disease. Patterns of practice in combining these treatments vary [4,5].

Ninety percent of vulvar cancers are squamous cell carcinomas (VSCC), which arise via high-risk human papilloma virus (hrHPV)-dependent and -independent pathways. The first pathway begins with the formation of high-grade squamous intraepithelial lesions (HSIL), while the other with a differentiated-type vulvar intraepithelial neoplasia (dVIN) [6]. Although p16-positivity considered a surrogate for hrHPV-positive cases is related with better survival [7], so far, there is no difference in therapeutic options for hrHPV-dependent and -independent disease [8]. Our recent review [9] of next-generation sequencing studies allowed recognition of the phosphatidylinositol 3-kinase (PI3K)/protein kinase B (AKT) signaling pathway as the main pathway deregulated in both hrHPV-induced and hrHPV-unrelated VSCC. Thus, novel therapeutic options targeting the PI3K/Akt pathway should be considered as potentially effective in VSCC patients’ management. The availability of biomarkers predictive for tumor aggressiveness would assist surgical decisions and enable individualized treatment. Unfortunately, clinically relevant, surgery-independent markers are still lacking for VSCC patients.

Extracting information on tumor-driving molecular changes in order to predict tumor development is one of the biggest challenges for translational oncology. The aim of this study was to advance the understanding of the VSCC biology by recognizing biological pathways that drive VSCC progression. For the analysis of protein expression, isobaric tags for relative and absolute quantitation (iTRAQ), a global mass spectrometry-based proteomic method was employed, which has so far proved successful in discovery-type experiments in identifying diagnostic cancer biomarkers [10]. Proteomic profiling was performed to characterize VSCC tumors and to identify protein markers that would indicate tumors more likely to progress. In silico investigation of proteomic data was used to provide hypotheses on the mechanism of VSCC progression based on biological pathways’ identification. Global techniques, such as iTRAQ, indicate potential biomarker candidates out of thousands of identified proteins. Therefore, consecutive steps of global data validation were applied in this study in search of protein with prognostic value in VSCC.

## 2. Results

### 2.1. Identification of Differentially Expressed Proteins Using iTRAQ

We applied the iTRAQ method to discover proteins with abundance alterations that are characteristic for the aggressive VSCC tumor phenotype, and to identify the molecular pathways underlying VSCC progression. The specimens included 16 VSCC tumor samples of patients that were found disease-free during the follow-up period (“d-fVSCC” group), 12 VSCC tumor samples obtained from patients who progressed (“progVSCC” group) and 14 normal vulvar tissue samples (controls). Among the d-fVSCC samples, eight were hrHPV-positive and seven were hrHPV-negative, while among the progVSCC samples, five were hrHPV-positive and seven were hrHPV-negative.

iTRAQ analysis identified 5509 proteins in this set of samples. The thresholds for differential expression was set to a protein level fold-change (FC) of >1.5 and <0.67, with a *p*-value of <0.05. With these thresholds, 586 differentially expressed proteins (DEPs) were identified between VSCCs (both d-fVSCC and progVSCC samples) and controls, of which 206 were increased and 380 were decreased in VSCCs. A set of hundreds of DEPs is difficult to interpret in terms of biological consequences/meaning of functional cellular alterations. Therefore, specialized tools were employed to translate identified DEPs into cellular processes: DEPs were submitted to Gene Ontology (GO) and Kyoto Encyclopedia of Genes and Genomes (KEGG) pathway analyses. GO and KEGG are considered the most comprehensive databases of molecular functions of genes and proteins and associated cellular processes. The results for the top 20 identifiers are provided in Tables S1 and S2, respectively.

**Figure 1:**
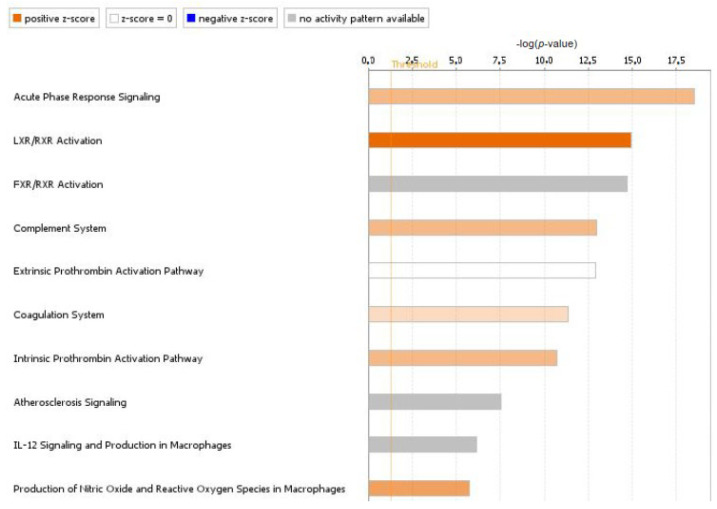
Top 10 canonical pathways enriched in progressed vulvar squamous cell carcinoma (progVSCC) compared to disease-free vulvar squamous cell carcinoma (d-fVSCC) tumors, identified by core analysis in Ingenuity Pathway Analysis (IPA). Top 10 canonical pathways enriched by IPA. The vertical orange line represents the threshold *p*-value (0.05) for pathway’s enrichment. The horizontal axis is the –log (*p*-value). Pathways predicted to be activated are in orange (based on the Z-scores, the activation scores determined by IPA), and grey bars relate to pathways with no activity pattern available.

We then compared the iTRAQ results obtained for d-fVSCC with those for progVSCC samples. The number of DEPs dropped down to 34, of which 27 were increased and 7 were decreased in levels. The GO and KEGG pathway analyses were conducted for DEPs in d-fVSCC and progVSCC samples. Interestingly, the identified set of significantly enriched GO biological process terms were different from those differentiating VSCCs from controls, and the immune response emerged as the most prevalent (Table S3). KEGG pathway analysis also revealed immune-related pathways among those dysregulated in VSCC (Table S4). DEPs with significant alterations in quantity were also submitted to core analysis in Ingenuity Pathway Analysis (IPA). The 10 canonical pathways most significantly affected in progVSCC compared to d-fVSCC samples (with a 1.2 threshold for the FC value), further emphasized the importance of inflammatory reactions that accompany VSCC progression (Figure 1 and Figure S1).

### 2.2. Verification by Parallel Reaction Monitoring

To verify our iTRAQ results in a larger sample set, we employed parallel reaction monitoring (PRM) to analyze 49 candidate protein biomarkers of VSCC (Table S5) 23 significantly differentially expressed proteins differentiated d-fVSCC from progVSCC tumors: 15 were increased and 8 decreased with FC > 1.5 and <0.9, respectively. Areas under receiver operating characteristics (ROC) curves (AUC) served to assess the feasibility of individual DEPs in distinguishing between d-fVSCC and progVSCC samples and between VSCC and normal vulvar tissues (Table 1).

### 2.3. Immunohistochemistry

From the dataset showing the list of 23 candidate biomarkers of VSCC progression verified with PRM analysis, two proteins—High Mobility Group AT-Hook 2 (HMGA2) and Proteinase 3 (PRTN3)—were analyzed by immunohistochemistry (IHC) in paraffin-embedded tissue specimens obtained from VSCC patients and from those with premalignant vulvar lesions (HSIL and dVIN). HMGA2 and PRTN3 proteins were selected for this analysis based on the difference in their abundance in d-fVSCC vs. progVSCC and in VSCC vs. control samples. Additionally, the AUC values indicated relatively high predictive values of both HMGA2 and PRTN3 to identify VSCC patients at risk of progression.

**Figure 2:**
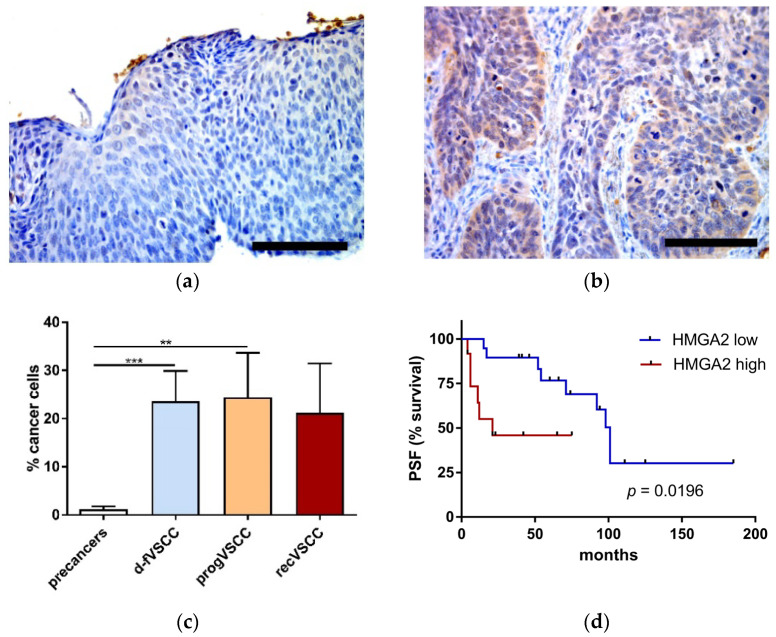
The percentage of HMGA2-positive neoplastic cells. Examples of immunohistochemical HMGA2 staining performed on tissue sections of high-grade squamous intraepithelial lesions (HSIL) (**a**) and progVSCC (**b**) tumor samples. Images were taken at 40× magnification. Scale bar, 100 μm. Comparison of the IHC results for premalignant lesions (HSIL; *n* = 44 and dVIN; *n* = 6), d-fVSCC (*n* = 19), progVSCC (*n* = 13) and recVSCC (*n* = 7) samples (**c**). Survival curve (time to progression) according to the relative numbers of HMGA2-positive cancer cells in primary VSCC tumors (*n* = 31) (**d**). Significant alternations are indicated by asterisks (**, *p*-value ≤ 0.01; ***, *p*-value ≤ 0.001). Abbreviations: PFS, progression-free survival; high and low, high and low proportion of HMGA2-positive cancer cells (≥30 and ≤10 cells, respectively).

HMGA2 staining was nuclear or both nuclear and cytoplasmic, and confined to neoplastic cells (Figure 2). In general, HMGA2 staining was weak in premalignant lesions and strong in VSCC tissues. Additionally, significantly higher relative numbers of HMGA2-positve neoplastic cells were seen in VSCCs than in vulvar pre-cancers (Figure 2c). The percentages of HMGA2-positve VSCC cells were not statistically significantly different in hrHPV-positive and -negative primary VSCC samples. Survival curves of the two groups of patients with primary VSCC tumors categorized by the percentage of HMGA2-positive cancer cells are shown in Figure 2d. The group with a high number of HMGA2-positive cells had a significantly shorter time to progression than patients with a low fraction of HMGA2-positive cells in the tumors. An increase of HMGA2 level may result from an inhibition of let-7 microRNA family members [11]. Our qPCR analysis confirmed a decrease of circulating let-7c levels in plasma of patients with premalignant lesions and VSCC patients compared to healthy volunteers (Figure S2).

**Figure 3:**
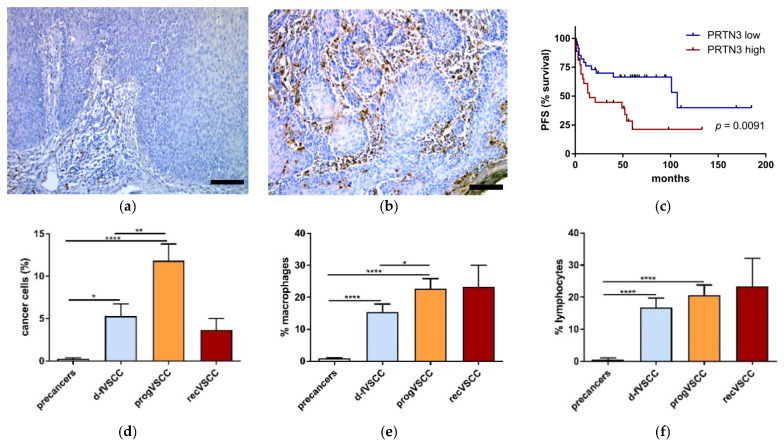
The results of PRTN3 immunostaining. Examples of immunohistochemical PRTN3 staining performed on tissue sections of HSIL (**a**) and VSCC tumors (**b**). Images were taken at 20x (**a-b**). Scale bar, 100 μm. The percent of PRTN3-positive neoplastic cells (**d**), macrophages (**e**), and lymphocytes (**f**) in vulvar pre-cancers (HSIL; *n* = 40 and dVIN; *n* = 5), d-fVSCC (*n* = 34), progVSCC (*n* = 31) and recurrent VSCC (*n* = 3) samples. Survival curve (time to progression) according to the proportion of PRTN3-positive cancer cells (**c**) in primary VSCC tumors (*n* = 64). Significant alternations are indicated by asterisks (*, *p*-value ≤ 0.05; **, *p*-value ≤ 0.01; ***, *p*-value ≤ 0.001; ****, *p*-value ≤ 0.0001). Abbreviations: PFS, progression-free survival; high and low, high and low proportions of PRTN3-positive cells.

PRTN3 staining was cytoplasmic and observed in neoplastic cells, macrophages, and lymphocytes (Figure 3). In general, the observed percentage of PRTN3-positive cells was lower among cancer cells than among the infiltrating non-neoplastic cells. Significantly higher relative numbers of PRTN3-positve cells were seen in VSCC than in vulvar premalignant lesions (dVIN and HSIL) (Figure 3d–f). Survival curves of the two groups of patients with primary VSCC tumors categorized by the number of PRTN3-positive cancer cells are shown in Figure 3c. Patients with higher numbers of PRTN3-positive cancer cells in the tumors had a significantly shorter time to progression than those with low numbers of these cells. The impact of PRTN3-positive macrophage or lymphocyte counts on survival was statistically insignificant.

### 2.4. PRTN3 Screening in VSCC Patients’ Blood

**Figure 4:**
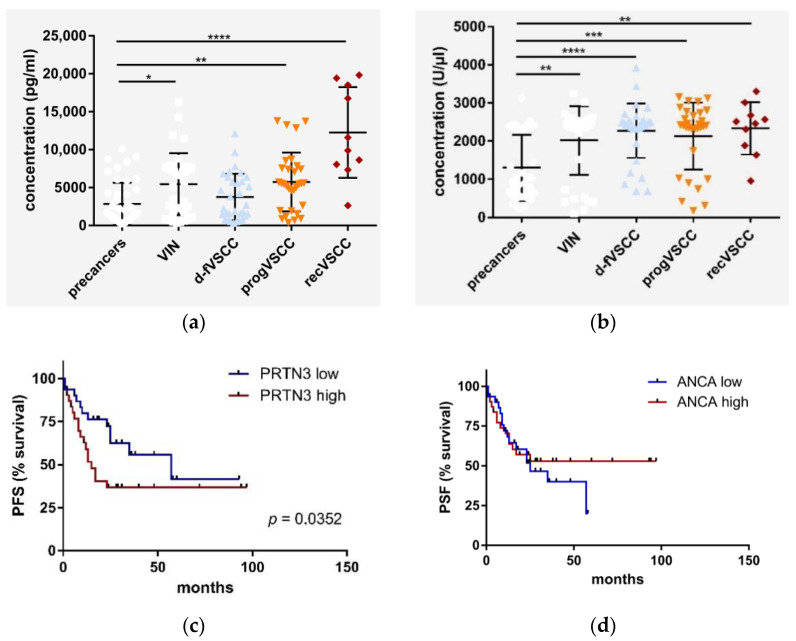
PRTN3 (**a**) and ANCA (**b**) serum levels. The results for the sera of 37 patients with vulvar pre-cancers (HSIL, *n* = 27; dVIN, *n* = 7), patients with primary (*n* = 64) and recurrent VSCC (*n* = 10) were compared to the control sera obtained from healthy women ((**c**), *n* = 44). Survival curves (time to progression) according to the serum levels of (**c**) PRTN3 and ANCA in patients with primary VSCC ((**d**), *n* = 64). Significant alternations are indicated by asterisks (*, *p*-value ≤ 0.05; **, *p*-value ≤ 0.01; ***, *p*-value ≤ 0.001; ****, *p*-value ≤ 0.0001). Abbreviations: PFS, progression-free survival; high, high protein level, i.e., above the cut-off value; low-protein level, i.e., below the cut-off value (i.e., the median of protein concentration).

PRTN3, secreted by neutrophils [12,13], belongs to neutrophil serine proteases, enzymes connecting innate immunity and coagulation [14]. Indeed, the complement and coagulation cascades pathway was at the top of the KEGG pathways identified in comparison of d-fVSCC and progVSCC samples by iTRAQ (Table S4, Figure S3). Therefore, to determine whether systemic alterations follow the phenomena observed in the analyzed tissues, the presence of PRTN3 was assessed by ELISA in the serum samples of patients with vulvar premalignant lesions and in VSCC patients, as well as in healthy women. The results of the measurement of PRTN3 as well as of anti-PRTN3 antibodies (ANCA, anti-neutrophil cytoplasmic antibodies) are shown in Figure 4a,b. Survival curves of the two groups of patients with primary VSCC tumors categorized by the PRTN3 and ANCA serum levels are shown in Figure 4c,d, respectively. The group of patients with primary VSCC with higher PRTN3 levels had a significantly shorter time to progression than the low PRTN3 group (15 vs. 57 months). The survival curves were not significantly different for patients with primary VSCC categorized by ANCA serum levels.

PRTN3 [14] is a modulator of human platelets’ function [15]. Platelets amplify inflammatory responses, and systemic alterations caused by cancer-related inflammation include an increase in both platelet and neutrophil counts as well as a decline in lymphocyte counts. Therefore, we sought to determine whether these cell counts as well as the neutrophil-to-lymphocyte ratio and the platelet-to-lymphocyte ratio in VSCC patients were also associated with disease progression, but no significant differences in those parameters between d-fVSCC and progVSCC patients were observed.

### 2.5. The Relevance of HPV Status of VSCC Tumors

The immune response identified as a key process driving VSCC progression could, at least partially, be explained by the consequences of HPV infection. Unexpectedly, the difference in relative numbers of HMGA2 and PTRN3-positive cells between hrHPV-positive and -negative primary VSCC samples was found to be statistically insignificant. We also compared the values of the analyzed basic hematological parameters in patients with hrHPV-positive versus hrHPV-negative VSCC tumors, and found no correlation. Similarly, no difference between PRTN3 and ANCA levels in the sera of these patients was observed.

**Figure 5:**
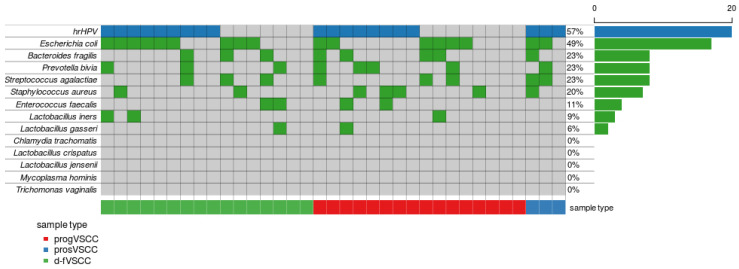
Distribution of bacterial species detected in VSCC samples. Each column corresponds to the individual tumor case (d-fVSCC, *n* = 16; progVSCC, *n* = 16 and prosVSCC, *n* = 3). Each row corresponds to an individual microorganism with its presence or absence depicted in green or grey, respectively. The results of genotyping of 13 vaginal microorganisms is related to hrHPV status of a given tumor with the virus presence or absence depicted in blue or grey, respectively. The values on the x-axis describe the number (0–20) and a percentage of the samples with the presence of a given microorganism.

This lack of association between these results and HPV status might be explained by the role of bacterial infections in VSCC. The *Staphylococcus aureus* infection pathway as second among the top KEGG pathways differentiating d-fVSCC and progVSCC proteomes (Figure S4), along with the microbicidal PRTN3 protein increase during the disease course, suggest that bacterial infections may be linked to aggressive VSCC phenotypes. Therefore, we performed PCR genotyping of 13 microorganisms known to contribute to vaginal dysbiosis on DNA isolated from 36 primary VSCC tumor samples. A pan-bacterial 16S rRNA gene DNA was found in all but one sample that eventually was excluded from the analysis due to poor DNA quality. The specific bacterial species (out of 11 bacterial species analyzed) were detected in 74% of samples (26/35) (Figure 5). The most common was *E. coli* (17/35), followed by *B. fragilis* (8/35), *P. bivia* (8/35), *S. agalactiae* (8/35), *S. aureus* (7/35), *E. faecalis* (4/35), *L. iners* (3/35), and *L. gasseri* (2/35).

## 3. Discussion

Individualization of the currently available treatment modalities is hampered by the lack of prognostic factors approved for clinical practice in VSCC [16]. The experimental path that we applied, from global proteomics to the quantitative analysis of specific proteins, was aimed to advance the understanding of the mechanisms underlying VSCC progression. A similar approach was previously confirmed by Sandberg et al. [17] while detecting protein alterations related to HPV infection in a set of VSCC tumors. The authors applied iTRAQ for the analysis of 14 specimens and global data were verified by IHC. Importantly, the proteins were selected for verification based on the relapse incidence in patients and regardless of the HPV status of their tumors. A high correlation between iTRAQ and IHC results was revealed for interferon-induced GTP-binding protein Mx1 (MX1) and LGMN (Legumain). Interestingly, MX1 was also among the DEPs identified in our study. This protein was found to be over 25-fold more abundant in VSCC compared to normal vulvar tissues according to the iTRAQ verification by PRM analysis.

In our study, we analyzed early-stage VSCC patients with a long-term follow-up after surgery. We identified the inflammatory response as the most prevalent constituent of the deregulated pathways in tumors of VSCC patients who progressed versus those of patients who were disease-free over a long follow-up time. This notion seems to be particularly justified as no immune-related pathways were found relevant when comparing VSCC tumors and normal vulvar tissues.

The immune infiltrate of VSCC and its precursor lesions by innate and adaptive immune cells, recently reviewed by Abdulrahman et al. [18], points towards the immunotherapeutic approaches as options to augment the current treatment modalities. The results of our modelling of VSCC tumorigenesis suggest that the innate immune response is a key attribute accompanying vulvar cancer progression. Local inflammatory reactions may result from a disturbed balance between the host and its commensal microbes and may promote cancer formation [19]. Neutrophil elastase (ELANE), PRTN3, and cathepsin G (CTSG) are the main neutrophil serine proteases (NSPs) released by activated neutrophils at the sites of inflammation, and are mediators of innate immune responses to microbial threats [14,20]. The preoperative values of neutrophil- and platelet-to-lymphocyte ratios were shown to be associated with nodal status, the most important prognostic factor in VSCC [21]. These ratios did not differ between d-fVSCC and progVSCC patients in our study, suggesting it is the activation of neutrophils and platelets during the disease course, rather than their increased numbers.

In our study, the Complement and coagulation cascades and *Staphylococcus aureus* infection were the two KEGG pathways the most significantly differentiating less aggressive and progressing VSCC tumors. In otherwise healthy women who contract genital tract infections, *S. aureus* is an infrequent but important pathogen [22,23]. Olejek et al. [24] found 90% of VSCC, as assessed prior to surgery, to contain pathogenic bacterial flora, including *S. aureus* species in 16% of the cases. It must be noted, however, that the *S. aureus* infection KEGG pathway may not be species-specific. Several microorganisms were previously associated with cervical cancer as well as risk for HPV infection and persistence [25]. Moreover, there is a trend toward an increased risk of cervical cancer progression in patients with vaginal dysbiosis [25]. Our preliminary PCR genotyping findings confirm the presence of pathogens, including *S. aureus*, within vulvar tumors.

The verification of the global protein analysis with targeted proteomics provided a list of reliable candidate biomarkers having “progression-classifier” properties, and these proteins need to be validated in future studies. In order to determine the histopathological context and cell localization of the two selected DEPs, we performed IHC analysis of VSCC tumors. HMGA2, the first verified candidate biomarker, belongs to the family of high-mobility group proteins, which alter chromatin structure and affect, either positively or negatively, the transcription of a variety of genes including genes related to the immune system functions [26,27]. HMGA2 overexpression can result from translocations at the 12q15 region [28] or down-regulation of let-7 microRNAs [5]. In our study, we have observed decreasing levels of circulating let-7c in plasma samples from patients with vulvar premalignant lesions and VSCC. Recently, HMGA2 was demonstrated to be expressed in VSCC tumors but not in the normal vulvar tissue [29]. In our study, HMGA2 tissue abundance was associated with the aggressive phenotype of VSCC.

The second verified protein, PRTN3 (myeloblastin) belongs to a family of NSPs, which digest matrix components in inflammation and, independently of their proteolytic activity, possess microbicidal properties [12]. Bacterial infections lead to neutrophil activation triggering their antimicrobial functions, involving neutrophil phagolysosomes and neutrophil extracellular traps (NETs) that encompass multiple proteases, including PRTN3 [30]. NETs can activate the alternative complement pathway and contribute to thrombus formation while in turn, platelets stimulate neutrophils to release NET with high-mobility group box 1 (HMGB1) expression on the platelets mediating the process [31]. In our study, circulating PRTN3 and ANCA, were found to be elevated in VSCC patients as compared to healthy female donors. This observation seems to be related to neutrophil or platelet activation rather than a change in their numbers. Both local and systemic abundance of PRTN3 found in VSCC patients suggests similarities in the mechanisms underlying VSCC and development of ANCA-associated vasculitis, a group of vasculitides characterized by neutrophil-rich inflammation of small vessels and the presence of circulating ANCAs. The analogies might include a key role of bacterial infection in these conditions.

PRTN3 has been proposed as a therapeutic target for example in chronic obstructive pulmonary disease, an inflammatory condition associated with neutrophilic inflammation [32]. Sivelestat, a selective ELANE and PRTN3 inhibitor [33] was shown to improve the mortality rates of patients with sepsis associated with acute respiratory distress syndrome and disseminated intravascular coagulation [34]. ELANE and PRTN3 along with CTSG are the NSPs stored in neutrophil granules and externalized during neutrophil activation at inflammatory sites [20]. In pathological conditions, these NSPs may interact with the complement pathway causing inefficient bacterial clearance [35]. Recently, Kam et al. [36] reported an inhibition of HMGB1 with sivelestat to be able to promote myelodysplastic syndrome cell death and alter innate immune responses via suppression of NFkB pathways. As HMGA2, another high-mobility group protein, also promotes the release of pro-inflammatory cytokines through regulation of the NFκB pathway [37], one might hypothesize that sivelestat could also block HMGA2, providing an additive benefit in VSCC therapy.

## 4. Patients and Methods

### 4.1. Patients and Samples

Material was obtained from patients undergoing surgery for vulvar premalignant lesions and VSCC (tissue and blood samples) and from healthy volunteers (blood) at the Maria Sklodowska-Curie National Research Institute of Oncology in Warsaw and at the Holycross Cancer Center in Kielce, Poland, between 2002 and 2019. Patients with VSCC at early (85 FIGO stage I, 7 FIGO stage II) and advanced stages (51 FIGO stage III, 2 FIGO stage IV) as well as patients with local recurrence (*n* = 17) were enrolled. Additionally, 14 patients who underwent routine plastic surgery of the reproductive organ at the Holy Family Hospital in Warsaw, Poland, between 2014 and 2015 were assigned to the control group. 

The databases of the Maria Sklodowska-Curie Institute-Oncology Center and the Holycross Cancer Center were retrospectively reviewed using the MedStream Designer application version 4.3.0.1 (Transition Technologies S.A., Poland) at the Onko.Sys platform to identify patients’ clinical (including results of vulvar swabs testing for bacterial infections) and follow-up data as well as preoperative platelet, neutrophil, and lymphocyte cell counts.

**Figure 6:**
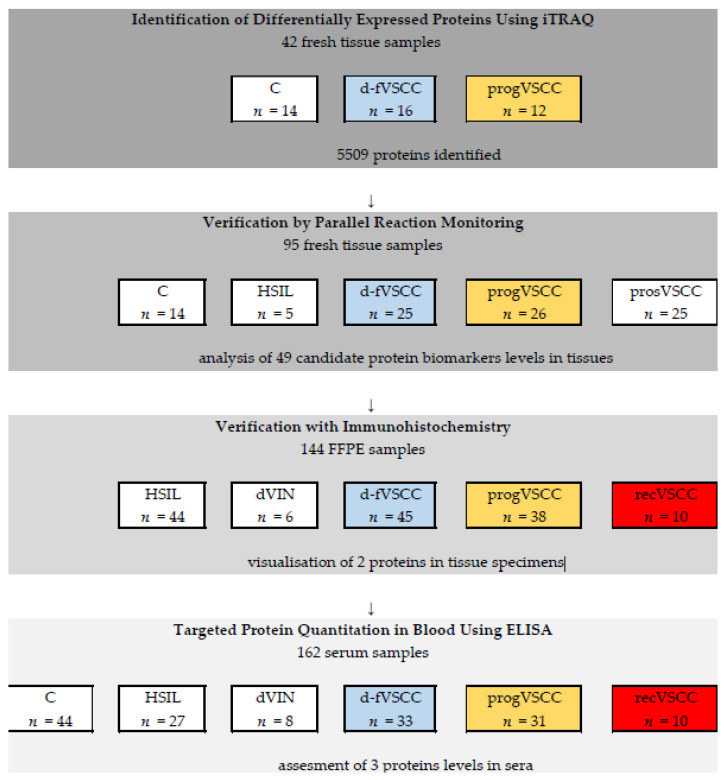
Study workflow from the discovery phase to protein selection and verification.

The numbers of samples, according to the stages of the study, are provided in Figure 6. Primary VSCC tumors samples were divided into two categories depending of the subsequent disease course. The first comprised tumors samples of patients that were found disease-free during the follow-up period (“d-fVSCC”) and the second—tumors samples obtained from patients who progressed (“progVSCC”). The progression was defined as local recurrence, regional LN recurrence, or cancer-related death. In addition, tumor samples obtained during vulvectomies of patients with primary VSCC enrolled prospectively (prosVSCC) and of patients with local VSCC recurrence (recVSCC) were included at the successive stages of the study. Finally, plasma samples were used for targeted protein quantitation in the blood of patients with vulvar premalignant lesions and VSCC. Normal vulvar tissue samples and sera of healthy female volunteers served as the control samples (C).

Basic characteristics of patients with primary VSCC (i.e., those from d-fVSCC, progVSCC, and prosVSCC groups) included are shown in Table 2.

### 4.2. Methods

#### 4.2.1. High Risk HPV Genotyping

The hrHPV status of tissue samples was determined, as described previously [36], using the AmpliSens^®^ HPV HCR-genotype-titre-FRT kit (InterLabService Ltd., Moscow, Russian Federation).

#### 4.2.2. Isobaric Tags for Relative and Absolute Quantitation (iTRAQ)

Forty-two (14 control, 16 d-fVSCC and 12 progVSCC) samples were subjected to deep proteomic analysis using iTRAQ method. Detailed descriptions of the iTRAQ methodology and resulting data processing can be found in the Text S1 (supplementary methods). Briefly, total cell lysates were obtained from approximately 100 mg of pulverized with the Microdismembrator II (B Braun Biotech International, Melsungen, Germany). Protein extract digestion and iTRAQ tag labeling was conducted using the 8-plex iTRAQ assay and iTRAQ Reagent and Buffer Kits (AB SCIEX, Foster City, CA, USA). A reference sample was used for normalization in each of the six independent 8-plex iTRAQ experiments.

#### 4.2.3. Protein Verification by Parallel Reaction Monitoring (PRM)

To screen for the presence of candidate proteins obtained in the iTRAQ experiment, a panel of 115 peptides from 49 proteins was chosen to verify putative prognostic proteins in 25 d-fVSCC and 26 progVSCC samples. Additionally, 14 controls, 5 HSIL, and 25 prosVSCC samples were included in the PRM analysis. Plasma samples obtained from 68 patients were analyzed with PRM assays to accurately quantitate 49 qualified proteins using stable-isotope-labeled internal peptide standards (3–5 peptides per protein). Detailed descriptions of the PRM methodology can be found in the Text S1.

#### 4.2.4. Histopathology and Immunohistochemistry

Fifty-one formalin-fixed paraffin-embedded (FFPE) samples, consisting of 44 HSIL, six dVIN, 50 d-fVSCC, 34 progVSCC and 10 recVSCC tumor specimens, were subjected to IHC analysis. The diagnosis of premalignant lesions of the vulva based on histologic criteria was confirmed using anti-p16 and p53 IHC as previously described [38] and in compliance with WHO 2020 recommendations [39].

IHC staining was performed on 4 μm FFPE tumor and reference tissue sections. The sections were deparaffinized with xylene and rehydrated in ethanol solutions. Heat-induced epitope retrieval was carried out in Target Retrieval Solution (Dako, Glostrup, Denmark) for 30 min at 96 °C. After cooling, the slides were treated for 5 min with an endogenous peroxidase blocker (Dako). The slides were incubated with polyclonal PRTN3 antibody (Sigma-Aldrich, Saint Louis, MO, USA; cat. No. HPA005938; 1:200 dilution) for 1 hour at RT or with polyclonal HMGA2 antibody (GenWay Biotech. Inc, San Diego, CA, USA; cat. No. GWB-78FFEC; 1:100 dilution) at 4 °C overnight. Subsequently, the slides were labelled with the EnVision FLEX+, Mouse, High pH Detection System (Dako). The color reaction product was developed with 3,3′-diaminobenzidine tetrahydrochloride (Dako) as a substrate, and nuclear contrast was achieved with hematoxylin counterstaining. The results of immunostainings for PRTN3 and HMGA2 were assessed by counting the percentage of positive cells per 10 high power fields (HPF).

Differentiation between lymphocytes and macrophages was carried out on the basis of their morphological features such as the size and shape of the cells as wells as cytological features of the nucleus assessed at high microscopic magnification (200–400×).

#### 4.2.5. Enzyme-Linked Immunosorbent Assays (ELISA)

Sera were obtained from the blood of 35 patients with vulvar premalignant lesions (HSIL, *n* = 27; dVIN, *n* = 8), 83 VSCC patients, and 44 healthy female volunteers. Concentrations of PRTN3 protein and antibodies against PRTN3 (i.e., anti-neutrophil cytoplasmic antibodies, ANCAs) were determined by applying Human PRTN3/Myeloblastin ELISA Kit (Sandwich ELISA) (LifeSpan BioSciences, Inc., Seattle, WA, USA; cat. No. LS-F21197) and ANCA-C (PR3) (Abnova, Taiwan; cat. No. KA1084) reagents kits, respectively, according to the manufacturers’ instructions. In both assays, the optical densities were read at 450 nm in Victor3™ Plate Reader (PerkinElmer, Waltham, MA, USA).

#### 4.2.6. Let-7c microRNA Quantification in Plasma

Total RNA was isolated from 200 µL of plasma using miRNeasy Mini Kit (Qiagen, Hilden, Germany), and miRNA was reverse transcribed using miScript II RT Kit (Qiagen) according to the manufacturer’s protocol. Expression levels of let-7c was analyzed by qRT-PCR using the miScript miRNA Arrays (Qiagen) according to the manufacturer’s protocol, as described previously [40]. Hsa-let-7c expression data were normalized to two reference miRNAs, hsa-miR-93-5p and hsa-miR-425-5p.

#### 4.2.7. Vulvovaginal Microorganism Detection Using PCR

Genomic DNA was isolated from 36 primary VSCC tumors (16 d-fVSCC, 17 progVSCC, and 3 prosVSCC samples) using the NucleoSpin Tissue kit (Macherey Nagel, Inc., Dueren, Germany), according to the manufacturer’s protocol. Real-time PCR was performed by using an QuantStudio 5 Real-Time PCR System (Thermo Fisher Scientific, Waltham, MA, USA). The reactions were carried out in a final volume of 20 μL containing 10 μL TaqMan™ Gene Expression Master Mix (Thermo Fisher Scientific) and 5 ng of template DNA. The reaction was performed in TaqMan Array 96 Plates (Thermo Fisher Scientific) containing primers and probes for 11 bacterial species, *Mycoplasma hominis* and *Trichomonas vaginalis* (listed in Supplementary Figure S3). The thermal cycling conditions were: 95 °C for 10 min, 45 cycles of 15 seconds at 95 °C, and 1 min at 60 °C. The microorganisms’ genotyping results were visualized as Oncoplots plotted using Maftools from R Bioconductor package [41].

#### 4.2.8. Statistical Analyses

iTRAQ data analysis was performed using the MScan, MSparky, MStat, and Diffprot software tools [42]. Differentially expressed proteins (DEPs) in d-fVSCC and progVSCC samples were searched (both for iTRAQ and PRM) by Wilcoxon test, with the statistical significance threshold set at *p*-value <0.05. The Benjamini–Hochberg procedure was used to adjust *p*-values due to multiple hypothesis testing. Overrepresentation of Gene Ontology (GO) terms [43] and Kyoto Encyclopedia of Genes and Genomes (KEGG) pathways [44] was conducted with GOstats version 2.42 [45], GO.db version 3.4.1, org.Hs.eg.db version 3.4.1, annotate version 1.54.0, and AnnotationDbi version 1.38.2 packages. For each GO/KEGG database, overrepresentation of the respective categories was tested among the top 10% of proteins, according to *p*-value. Areas under receiver operating characteristics (ROC) curves (AUC) used to assess the performance of DEPs in PRM analysis, along with GO and KEGG overrepresentation, were calculated in the R environment version 3.4.1 (http://www.R-project.org). For canonical pathways (and network analyses) DEPs were submitted to Ingenuity Pathway Analysis (Ingenuity Systems, USA; http://www.ingenuity.com, accessed on 9 August 2019).

The Kruskal–Wallis test followed by Wilcoxon multiple pair-wise comparisons was used to test the significance of differences in protein expression between the tissue and serum sample groups. The Benjamini–Hochberg procedure was used to adjust *p*-values due to multiple hypothesis testing. *p*-value < 0.05 was considered significant. Median follow-up time of the enrolled patient was determined from the date of surgery to the date of death or the date of the last interview was registered. Association between the protein content of selected proteins in both tumor and blood samples with time to progression of the disease in patients with primary VSCC was determined using Cox proportional hazard model implemented in the survival package (version 2.41-3) in the R environment.

The results were visualized using GraphPadPrism (San Diego, CA, USA). 

## 5. Conclusions

In summary, the biological insight from the study points at inflammation as a key event during VSCC tumorigenesis. Indirect but numerous lines of evidence suggest that changes in the genital microbial flora disturbances may be the causative agents in vulvar cancer-related inflammation. Although vulvar carcinoma does not present a diagnostic challenge, methods to predict the disease course in early-stage VSCC patients are missing. In early-stage VSCC, HMGA2 and PRTN3 should be considered as potential predictive markers and putative therapeutic targets. Increased abundance of HMGA2 and PRTN3 in aggressive VSCC was found to be independent of the hrHPV status of the tumors. This further suggests that bacterial rather than viral infection status should be considered in the development of targeted therapies in VSCC. Prevention and control of bacterial infections are anticipated to become a valuable addition to current cancer treatment modalities [46,47]. Finally, several similarities in the biology of AVV provides grounds for the assumption that VSCC patients might benefit from treatments currently applied for AVV and other neutrophilic diseases.

## Figures and Tables

**Table 1:** The list of 23 differentially expressed proteins (DEPs) identified in the iTRAQ experiment and confirmed using PRM analysis.

Protein	d-fVSCC vs. progVSCC				VSCC vs. Normal Vulvar Tissues			
	*p*-Value	adjp	FC	AUC	*p*-Value	adjp	FC	AUC
HMGA2	0.0063	0.0824	14.4	0.73	0.0059	0.0095	45.5	0.76
ANO1	0.0068	0.0824	3.37	0.73	0.6177	0.6312	1.80	0.55
PRTN3	0.0117	0.0981	2.75	0.71	<0.0001	<0.0001	13.3	0.92
UBE2C	0.0070	0.0824	2.72	0.73	0.0001	0.0002	8.89	0.87
KRT18	0.0220	0.0981	2.48	0.70	<0.0001	0.0001	8.18	0.91
S100A12	0.0160	0.0981	2.44	0.71	<0.0001	<0.0001	12.0	0.92
PTX3	0.0359	0.1211	2.31	0.68	0.0535	0.0662	2.68	0.68
RUNX1	0.0192	0.0981	2.06	0.70	<0.0001	0.0001	6.73	0.90
PADI2	0.0192	0.0981	1.89	0.70	<0.0001	<0.0001	3.72	0.88
ISG15	0.0273	0.1069	1.88	0.69	<0.0001	<0.0001	35.4	0.94
ABCC10	0.2133	0.3992	1.75	0.61	0.0068	0.0106	5.55	0.75
IFIT3	0.0811	0.1907	1.74	0.65	<0.0001	<0.0001	16.5	0.91
IDO1	0.8737	0.9126	1.68	0.52	0.0001	0.0001	17.4	0.88
PTPMT1	0.2067	0.3992	1.57	0.61	0.0815	0.0957	1.72	0.67
MX1	0.2550	0.4279	1.50	0.60	<0.0001	<0.0001	25.5	0.93
KRTDAP	0.6183	0.7647	0.88	0.54	0.0034	0.0057	0.49	0.77
OGN	0.0670	0.1852	0.81	0.66	<0.0001	<0.0001	0.20	0.91
A2ML1	0.1499	0.3202	0.76	0.63	0.0329	0.0429	0.65	0.70
EDEM2	0.2844	0.4435	0.74	0.59	0.1983	0.2273	1.85	0.62
ABI3BP	0.0230	0.0981	0.71	0.69	<0.0001	<0.0001	0.16	0.92
PRELP	0.0160	0.0981	0.70	0.71	<0.0001	<0.0001	0.26	0.93
CSTA	0.2925	0.4435	0.69	0.59	0.4850	0.5066	1.12	0.57
SPRR3	0.0811	0.1907	0.62	0.65	0.3964	0.4234	2.07	0.58

Abbreviations: adjp, adjusted *p*-value; FC, fold change of differential protein content; AUC, area under receiver operating characteristics (ROC) curves.

**Table 2:** Demographic and clinical characteristics of the patients with primary VSCC. Numbers of samples are provided for the consecutive phases of the study.

Characteristic		Stage of the Study			
		iTRAQ Analysis	Verification by PRM	IHC Analysis	ELISA Serum Testing
Age–years	Median	70.0	70.8	70.4	72.6
	Range	45.3–93.9	45.3–94.2	37.1–93.9	41.8–87.9
FIGO stage	I	19	46	54	35
	II	2	5	3	4
	III	7	24	29	22
	IV	0	1	0	3
Histological grade	G1	12	23	34	17
	G2	9	28	37	28
	G3	6	12	12	6
	N/D	1	13	6	13
hrHPV tumor status	positive	13	38	36	31
	negative	15	27	32	6
	N/D	0	11	18	27
Disease progression	d-fVSCC	16	25	50	33
	progVSCC	12	26	34	31
	prosVSCC	0	25	3	0

Abbreviations: iTRAQ, Isobaric tags for relative and absolute quantitation; PRM, parallel reaction monitoring; N/D, not determined.

## Data Availability

Proteomic data is uploaded to a public proteomic repository and will be shared upon request to the corresponding author.

## Supplementary Materials

The following are available online at https://www.mdpi.com/2072-6694/13/1/27/s1, Text S1: Supplementary Methods, Figure S1: Overlapping canonical pathways identified by core analysis in Ingenuity Pathway Analysis (IPA) that were significantly enriched in the dataset, Figure S2: Let-7c levels in plasma samples of patients with VIN (*n* = 21), primary VSCC (*n* = 34) and recurrent VSCC (recVSCC, *n* = 2) as compared to control plasma samples obtained from healthy women (C, *n* = 15), Figure S3: KEGG pathway view on proteomic data (hsa04610), Figure S4: Staphylococcus aureus infection (hsa5150) KEGG pathway, Table S1: The top 20 overrepresented GO term for DEPs in VSCC and normal vulvar tissues, Table S2. The top 20 KEGG pathways in VSCC and normal vulvar tissues, Table S3: The top 20 overrepresented GO terms for DEPs in progVSCC versus d-fVSCC samples, Table S4: The top 20 KEGG pathways for DEPs overrepresented in progVSCC versus d-fVSCC samples, Table S5: Candidate VSCC biomarkers examined in PRM analysis.
