# Supplementary material for: Inflammatory Proteins HMGA2 and PRTN3 as Drivers of Vulvar Squamous Cell Carcinoma Progression
Source: Cancers (Basel). 2020 Dec 23;13(1):27. doi: 10.3390/cancers13010027 (PMC7793473; doi:10.3390/cancers13010027)
Supplement: Supplementary file 1 [file cancers-13-00027-s001.pdf]

Supplementary Materials: Inflammatory Proteins HMGA2 and PRTN3 as Drivers of Vulvar Squamous Cell Carcinoma Progression

Agnieszka Fatalska, Natalia Rusetska, Elwira Bakuła-Zalewska, Artur Kowalik, Sebastian Zięba, Agnieszka Wroblewska, Kamil Zalewski, Krzysztof Goryca, Dominik Domański and Magdalena Kowalewska

Text S1: Supplementary Methods

iTRAQ (Isobaric Tags for Relative and Absolute Quantitation)

Cell Lysis

The samples were snap-frozen in liquid nitrogen immediately after collection and stored at −70°C before pulverization (with the Microdismembrator II, B Braun Biotech International, Melsungen, Germany). Forty-two (14 control, 16 d-fVSCC and 12 progVSCC) samples were subjected to deep proteomic analysis. Total cell lysates were obtained from approximately 100 mg of pulverized patient tissue samples.

The frozen pulverized tissue samples were resuspended in 300 µl of cold Lysis Buffer (1% sodium deoxycholate (NaDOC) in 25 mM HEPES (4-(2-hydroxyethyl)-1-piperazineethanesulfonic acid), boiled for 5 min at 100 °C, and cooled to room temperature (RT) on ice. Next, nucleic acids were degraded with 30 µL of benzonase nuclease (2.5 U/µL in 25 mM HEPES) at 4 °C for 30 min. Samples were then centrifuged twice at 12,000 × g for 10 min at 4 °C and the supernatants were stored at −80 °C. Protein concentrations were determined using the Pierce (Appleton, WI, USA) Bicinchoninic Acid (BCA) Assay Kit according to the manufacturer's instructions, for the 96-well plate format, in duplicate, at two different sample dilutions (ThermoFisher Scientific, Waltham, MA, USA).

Sample Protein Extract Digestion and iTRAQ Labeling

Protein extract digestion and iTRAQ tag labeling was conducted using the 8-plex iTRAQ assay and iTRAQ Reagent and Buffer Kits (AB SCIEX, Framingham, MA, USA). 100 µg of proteins from each lysate was made up to 20 µl with Lysis Buffer. 20 µl of Dissolution Buffer (triethylammonium bicarbonate (TEAB), pH 8.5) and, for protein denaturation, 1 µL of 2% SDS (sodium dodecyl sulfate) was added to each sample. To reduce disulfide bonds, 2 µL of 50 mM TCEP (tris(2-carboxyethyl)phosphine) was added and the sample was incubated for 1 hour, at 60 °C. Cysteines were blocked by addition of 1 µL of 200 mM methyl methanethiosulfonate (MMTS) and incubation for 10 min at RT. Five µL of Dissolution Buffer was added, followed by 10 µL (8 µg) of trypsin, and incubated for 16 hours at 37 °C to digest proteins. Next, 11 µL of Dissolution Buffer was added to each sample. Each 8-plex iTRAQ reagent tag was dissolved in 115 µL of isopropanol, then added to each individual digest, and incubated for 2 hours at RT. The eight labeled samples were combined, the amount of isopropanol reduced using a SpeedVac, and desalted using Solid Phase Extraction cartridges (SPE Oasis HLB 1cc (10 mg), Waters, Milford, MA, USA): diluted in 0.5% formic acid (FA) into two 1 mL fractions and centrifuged (5 min at 10,000 × g) to remove precipitated NaDOC, loaded onto two cartridges, washed with 1 mL of LC-MS water, and eluted with 300 µL of 75% acetonitrile (ACN), 0.1% FA. Samples were dried using a SpeedVac and stored at −80 °C till high-pH fractionation.

A Reference Sample for iTRAQ Analysis

Equal amounts of digested lysates (100 µg of proteins from each lysate) from 4 control, 4 d-fVSCC and 4 progVSCC samples were pooled to create a reference sample. After pooling, this sample was processed as described above, i.e., the same way as the studied samples. The reference sample was used for normalization in each of the six independent 8-plex iTRAQ experiments.

High-pH Reversed-Phase Fractionation

For extensive peptide fractionation, 755 µg (dissolved in 10 mM ammonium hydroxide) of the iTRAQ-labeled tissue digest was separated with an H-Class Waters UHPLC system at high-pH (10 mM ammonium hydroxide, pH 10) on a reversed-phase column (Waters XBridge Peptide BEH C18 Column: 130 Å, 5 µm, 4.6 mm × 250 mm) at a 1 mL/min flow rate and a gradient of 3–55% acetonitrile (ACN) over 60 min followed by a washing step (10 min at 90% ACN) and column equilibration (15 min at 3% ACN). Sixty 1 mL fractions were collected, dried completely using a SpeedVac, dissolved in 50 µL of 0.1% FA and 20 µL was used for nano-LC-MS/MS analysis.

LC-MS/MS Analysis

A Waters nanoACQUITY UPLC system was used for peptide separation prior to MS/MS analysis. Mobile phase A consisted of 0.1% FA and mobile phase B was ACN/0.1% FA. 20 µL of each fraction was injected onto a reversed-phase trapping column (180 µm × 20 mm, C18, 5 µm, ACQUITY UPLC Symmetry, Waters) using mobile phase A. Peptides were transferred to a reversed-phase analytical column (75 µm × 250 mm, nanoACQUITY UPLC BEH130 C18 Column, 1.7 µm, Waters) and separated using a 250 nL/min flow rate and a gradient from 3–33% mobile phase B over 150 min. The column was directly coupled to the ion source of an Q-Exactive Orbitrap mass spectrometer (Thermo Fisher Scientific) which worked in the regime of data-dependent MS to MS/MS switch, with the following criteria: positive polarity mode (2–3 kV), capillary temperature of 250 °C, HCD fragmentation (NCE: 27), isolation width 1.2 Th, one MS scan (300–2000 m/z, 70k resolution, 60 ms max fill time, 1e6 AGC), followed by a maximum of 12 MS/MS scans (17.5k resolution, 60 ms max fill time, 5e5 AGC), with a dynamic exclusion of 30 s and profile mode acquisition.

Qualitative MS/MS Data Processing and iTRAQ Quantitative Analysis

Data processing was performed as previously described [1]. Briefly, Mascot Distiller (version 2.5.1.0, Matrix Science, London, UK) was used to pre-process MS/MS data with default value settings for iTRAQ labeling. This included merging of redundant spectra and removal of noisy spectra. Next, a two-step database search procedure was carried out [2]. A first, less restrictive database search of the data was conducted using the MASCOT search engine and the Swiss-Prot Homo sapiens database (from 2015) which enables the calculation of MS and MS/MS measurement errors and recalibration of the data for a repeated stringent MASCOT search. The initial search parameters were set as follows: enzyme, trypsin; fixed modification, cysteine modification by MMTS as well as iTRAQ labeling of the *N*-terminus of peptides and of lysine side chains; variable modifications—oxidation (M); max missed cleavages—1, peptide tolerance of 100 ppm, MS/MS tolerance of 0.2 Da. The data were calibrated and filtered using the MScan program with a queries threshold of 10. The merged file of all the fractions was then again searched using MASCOT against the Swiss-Prot database supplemented with a decoy database of randomized sequences mixed in, enabling the calculation of the false discovery rate (FDR). This procedure provided q-value estimates for each peptide spectrum match (PSM) in the dataset. All PSMs with q-values >0.01 were removed from further analysis. Additional search parameters to those mentioned above now included a peptide tolerance of 6 ppm and a fragment ion tolerance of 0.2 Da. Further data filtration was performed using MScan with the following criteria: at least two peptide observations per protein, proteins that matched the same set of peptides were clustered into one protein group, and proteins identified by a subset of peptides from another protein were removed from the analysis. MS/MS spectra of peptides meeting the above acceptance criteria were subjected to the quantitative analysis step to obtain a list of differentially expressed proteins as indicated by the iTRAQ reporter tags. The differentially expressed proteins list with estimation of statistical significance of a single protein ratio was acquired using the in-house program Diffprot as described previously [2].

Verification by Parallel Reaction Monitoring (PRM)

Using total protein digests from each sample, a targeted proteomic method, parallel reaction monitoring (PRM) was employed to verify the differentially expressed proteins. Three to five unique peptides of each protein of interest were selected. To screen for the presence of candidate proteins obtained in the iTRAQ experiment, a panel of 115 peptides from 49 proteins was chosen to verify putative prognostic proteins in 25 d-fVSCC, and 26 progVSCC samples. Additionally, 14 controls, 5 HSIL and 25 prosVSCC samples were included in the PRM analysis. Plasma samples obtained from 68 patients were analyzed with PRM assays to accurately quantitate 49 qualified proteins using stable-isotope standards (3–5 peptides per protein).

Selection of Peptides for PRM

The selection of peptides for PRM was performed according to the criteria described previously [3]. Briefly, tryptic peptides best suited for targeted analysis were selected using databases such as: UniProt, PeptideAtlas, SwissProt-Expasy, NCBI BLASTp, and the NCBI SNP database with the following criteria: unique peptide sequence to the targeted protein, highly observed in spectral databases, peptide length does not exceed 21 amino acids, avoided easily chemically modifiable residues and sequences prone to modifications, peptides with missed cleavages and low efficiency of digestion were excluded, peptides containing high frequency single-nucleotide polymorphisms were excluded, and peptides containing reported post-translational modifications or with known biological features affecting their accurate measurement were excluded.

For the stable-isotope dilution-PRM (SID-PRM) analysis, the selected peptide sequences were synthesized as stable-isotope labeled standard (SIS) peptides, using isotopically labeled amino acids on the C-terminus: Arg $^{13}C_6$; $^{15}N_4$ (98% isotopic enrichment) or Lys $^{13}C_6$; $^{15}N_2$ (98% isotopic enrichment) by JPT Peptide Technologies GmbH (Berlin, Germany) as SpikeTides_L.

Trypsin Digestion of Patient Tissue Lysates for Verification

From each cell lysate (obtained as described above in the discovery phase), 100 µg of proteins was transferred into a microplate well into the already added 1% NaDOC in 25 mM ammonium bicarbonate (AmmBic), to a final volume of 105 µL, followed by an addition of 8 µL of 3.75% NaDOC in 100 mM AmmBic. Disulfide bonds were reduced by the addition of 4 µL of 50 mM tris(2-carboxyethyl)phosphine (TCEP) in 100 mM AmmBic, and incubation at 60 °C for 30 min. To block cysteine groups, 4 µL of 100 mM iodacetamide (IAA) in 100 mM AmmBic was added and incubated at 37 °C for 30 min. Next, 4 µL of 100 mM dithiothreitol (DTT) in 100 mM AmmBic was added and incubated at 37 °C for 30 min to quench extra IAA. To digest proteins, 10 µL (4 µg) of trypsin (ratio: 25:1 protein: enzyme) was added and the plate was incubated for 16 hours at 37 °C. 50 µg of digest from each sample was transferred into a new microplate well, 40.3 µL of a SIS mixture (115 SIS peptides balanced and mixed together) were added, and NaDOC was precipitated by addition of 42.2 µL of 1% FA. The precipitate was removed by centrifugation at 3000 × g for 5 min., and the supernatant was cleaned on an SPE plate (Waters, Oasis HLB, micro-elution plate), by adding to 350 µL of 0.2% FA followed by washing with 500 µL of LC-MS water and eluting with 100 µL of 55% ACN, 0.1% FA. Each sample was dried on a Speed-Vac, dissolved in 50 µL of 0.1% FA and stored at −80 °C till PRM analysis.

Digestion of Plasma Samples

Five µL of plasma was transferred into 50 µL of 9 M urea. To reduce disulfide bonds 9 µL of 50 mM TCEP in 100 mM AmmBic was added and incubated at 37 °C for 30 min with shaking. Next, to block cysteine groups, 5 µl of 100 mM IAA in 100 mM AmmBic was added and incubated with shaking at 37 °C for 10 min. To quench IAA, 2.5 µL of 100 mM DTT in 100 mM AmmBic was added and incubated with shaking at 37 °C for 30 min. After this time, 428.5 µL of 100 mM AmmBic was added to dilute urea to 0.9 M, and 35 µL (14 µg) of trypsin in 100 mM AmmBic (ratio 25:1 protein: enzyme) was added and incubated at 37 °C for 16 hours. To each sample, SIS peptides were added and the sample was

desalted using SPE cartridges (Waters, Oasis HLB 1cc (10 mg)), as follows: the samples were diluted in 0.1% FA, loaded, washed with 1 mL of LC-MS water, and eluted with 200 μL of 55% ACN, 0.1% FA, and finally dried using a SpeedVac and kept at −80 °C prior to PRM analysis.

PRM nanoLC-MS Analysis

The Q-Exactive nanoLC-MS system was used as for the iTRAQ analysis described above except the instrument was used in PRM mode: in multiplexed-mode for natural and SIS peptides (MSX: 2), isolation width of 1.0 Th, with a resolution of 35k (tissue samples) and 70k (plasma), a 60/120 ms fill time, and AGC of 1e6. Data were analyzed in Skyline with up to 10 selected ion fragments picked according to a spectral library generated on a pure mixture of SIS peptides which generated the highest signals and which were selected for each peptide that were free of signal interferences for both the endogenous and heavy (SIS) peptides. For the SID-PRM analysis the quantity of the endogenous peptide is reported as the Peak Area Ratio (PAR) which is the sum of the peak areas of all transitions for the endogenous peptide divided by the sum of the peak areas of transitions of its heavy standard. The addition of equivalent amounts of standard peptides to the analyzed samples and the calculation of PAR enables the normalization of natural peptide relative abundance between samples in terms of MS signal fluctuations and post-digestion sample processing differences. NanoLC-PRM-MS analysis was carried out with 5 μg of digested proteins (1 μg/μL in 0.1% FA), with three blank (0.1% FA) runs in between every sample. All PRM data were processed using the Skyline Ver. 3 software with default values for peak integration. All integrated peaks were manually inspected to ensure correct peak detection and accurate integration. All peptides were targeted using 5 to 10 ion pairs per peptide that were interference free.

References

Bakun, M.; Niemczyk, M.; Domanski, D.; Jazwiec, R.; Perzanowska, A.; Niemczyk, S.; Kistowski, M.; Fabijańska, A.; Borowiec, A.; Paczek, L.; et al. Urine proteome of autosomal dominant polycystic kidney disease patients. *Clin. Proteom.* **2012**, *9*, 13, doi:10.1186/1559-0275-9-13.

1. Malinowska, A.; Kistowski, M.; Bakun, M.; Rubel, T.; Tkaczyk, M.; Mierzejewska, J.; Dadlez, M. Diffprot — software for non-parametric statistical analysis of differential proteomics data. *J. Proteom.* **2012**, *75*, 4062–4073, doi:10.1016/j.jprot.2012.05.030.
2. Mohammed, Y.; Domański, D.; Jackson, A.M.; Smith, D.S.; Deelder, A.M.; Palmblad, M.; Borchers, C.H. PeptidePicker: A scientific workflow with web interface for selecting appropriate peptides for targeted proteomics experiments. *J. Proteom.* **2014**, *106*, 151–161, doi:10.1016/j.jprot.2014.04.018.

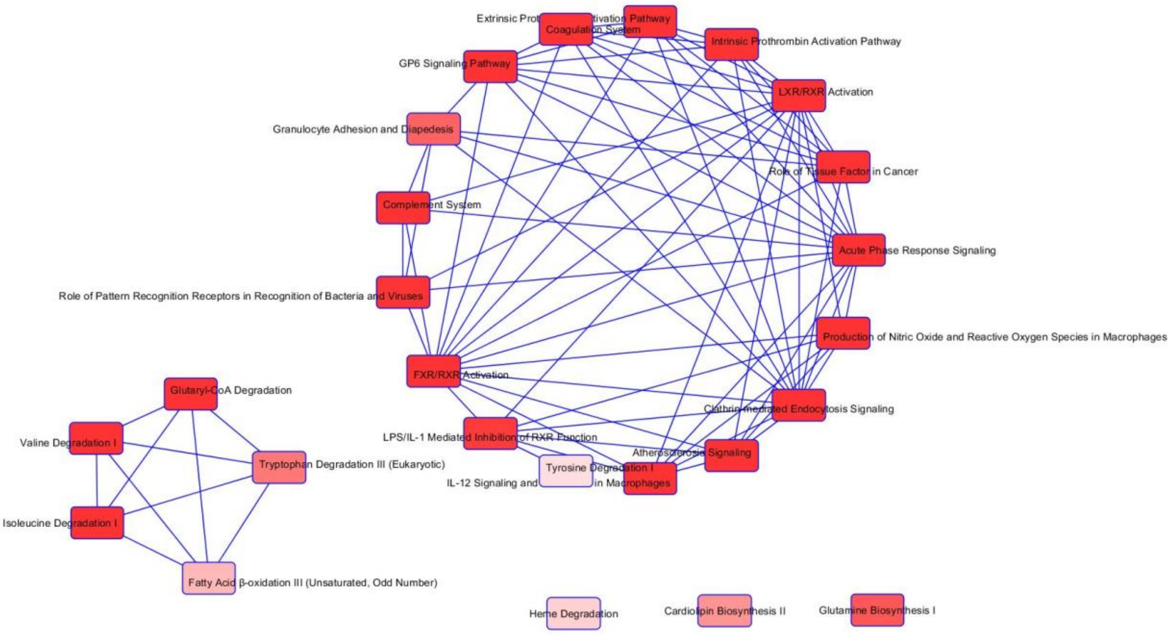

Figure S1. Overlapping canonical pathways identified by core analysis in Ingenuity Pathway Analysis (IPA) that were significantly enriched in the dataset. Nodes correspond to canonical pathways whereas links correspond to the molecules shared between two pathways (no line means no shared molecules between two pathways). The color intensity of nodes corresponds to the −log *p*-value (a more intense color indicates a larger −log *p*-value).

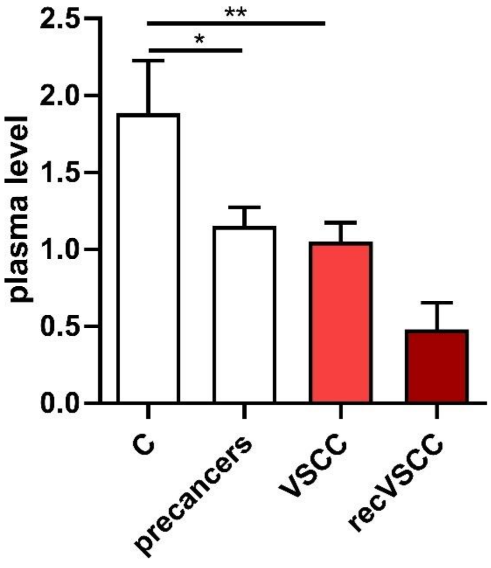

Figure S2. Let-7c levels in plasma samples of patients with VIN (n = 21), primary VSCC (n = 34) and recurrent VSCC (recVSCC, n = 2) as compared to control plasma samples obtained from healthy women (C, n = 15). Significant alternations are indicated by asterisks (*, *p*-value ≤0.05; **, *p*-value ≤ 0.01).

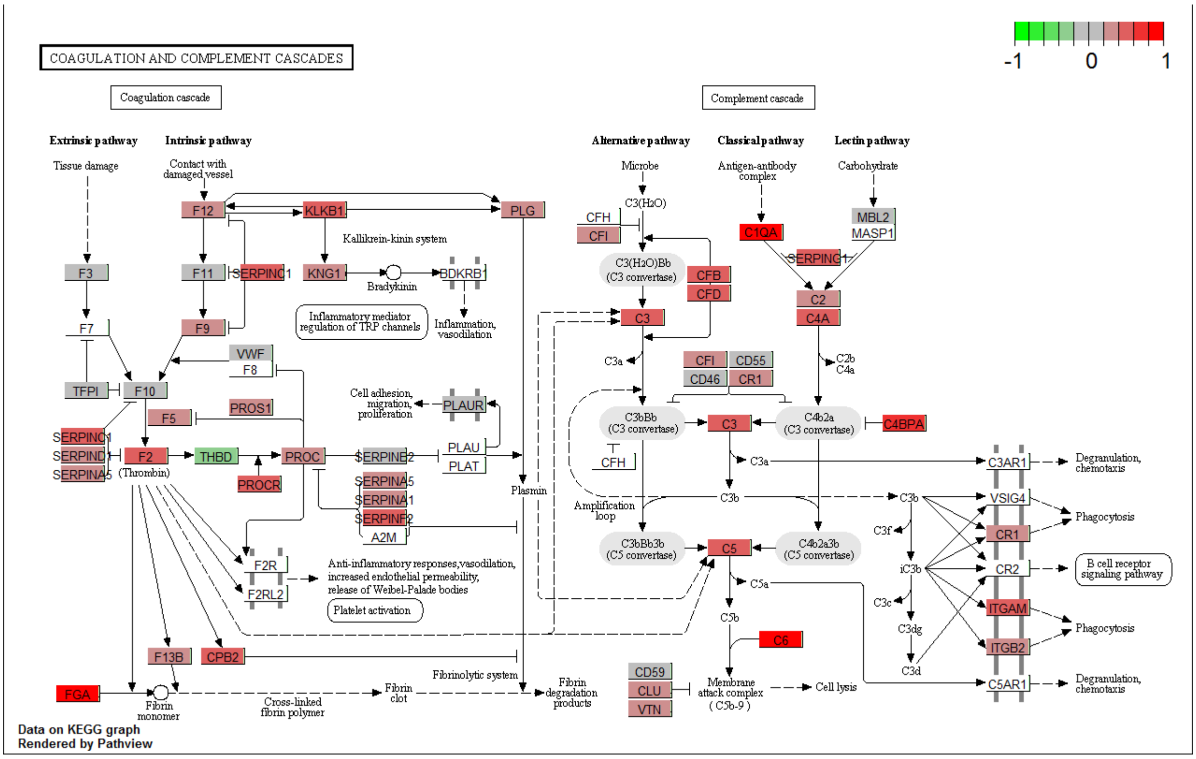

Figure S3. KEGG pathway view on proteomic data (hsa04610). Red and green indicate an increase and decrease of DEP levels, respectively, in progVSCC samples compared to d-fVSCC tumors (log2 fold-change, $p < 0.05$). DEPs, differentially expressed proteins; KEGG, Kyoto Encyclopedia of Genes and Genomes.

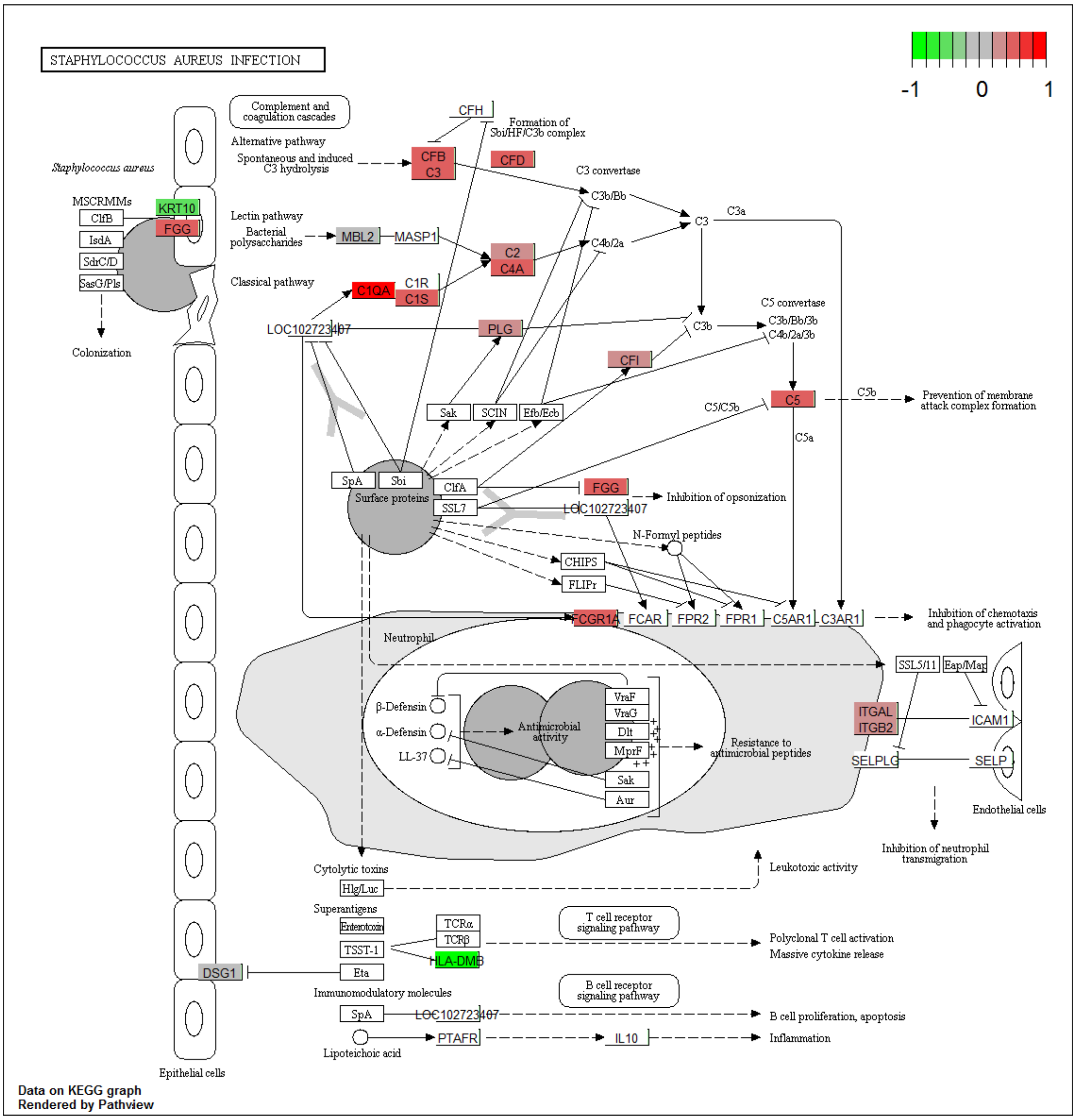

Figure S4. Staphylococcus aureus infection (hsa5150) KEGG pathway. Red and green indicate up- and down-regulation of DEPs, respectively, in progVSCC samples compared to d-fVSCC tumors (log2 fold-change, *p*-value < 0.05). DEPs, differentially expressed proteins; KEGG, Kyoto Encyclopedia of Genes and Genomes.

Table S1. The top 20 overrepresented GO term for DEPs in VSCC and normal vulvar tissues.

Name	Term	*p*-value	Adjp
GO:0051234	establishment of localization	0.000204	0.015108
GO:0048522	positive regulation of cellular process	0.000161	0.013502
GO:0007275	multicellular organism development	3.2×10^{-6}	0.000937
GO:0032502	developmental process	1.33×10^{-5}	0.002496
GO:0050896	response to stimulus	4.46×10^{-7}	0.000163
GO:1902578	single-organism localization	1.66×10^{-8}	1.75×10^{-5}
GO:0010646	regulation of cell communication	4.12×10^{-5}	0.005746
GO:0023051	regulation of signaling	8.8×10^{-5}	0.009377
GO:0016192	vesicle-mediated transport	0.000358	0.023101
GO:0050790	regulation of catalytic activity	0.000107	0.01025
GO:0032879	regulation of localization	0.000529	0.029662
GO:0065008	regulation of biological quality	9.32×10^{-6}	0.002025
GO:0050789	regulation of biological process	0.000145	0.012879
GO:0051239	regulation of multicellular organismal process	4.75×10^{-5}	0.006477
GO:0023052	signaling	2.06×10^{-5}	0.003451
GO:0007166	cell surface receptor signaling pathway	0.000941	0.047563
GO:0032270	positive regulation of cellular protein metabolic process	0.000292	0.01967
GO:0009967	positive regulation of signal transduction	0.000323	0.021291
GO:0043436	oxoacid metabolic process	0.000438	0.02541
GO:0051674	localization of cell	0.001042	0.049297

Abbreviations: GO—Gene Ontology; adjp—adjusted *p*-value.

Table S2. The top 20 KEGG pathways in VSCC and normal vulvar tissues.

Name	Path	*p*-value	Adjp
4610	Complement and coagulation cascades	3.28×10^{-10}	5.74×10^{-8}
3050	Proteasome	2.23×10^{-5}	0.001616
5150	Staphylococcus aureus infection	2.77×10^{-5}	0.001616
5416	Viral myocarditis	0.000106	0.004651
5310	Asthma	0.00015	0.005239
4612	Antigen processing and presentation	0.000388	0.011306
330	Arginine and proline metabolism	0.000773	0.019327
5020	Prion diseases	0.001122	0.02455
340	Histidine metabolism	0.003227	0.062742
4514	Cell adhesion molecules (CAMs)	0.003682	0.064431
300	Lysine biosynthesis	0.013624	0.200781
5332	Graft-versus-host disease	0.014277	0.200781
630	Glyoxylate and dicarboxylate metabolism	0.01727	0.200781
480	Glutathione metabolism	0.018377	0.200781
640	Propanoate metabolism	0.020661	0.200781
4940	Type I diabetes mellitus	0.021644	0.200781
5320	Autoimmune thyroid disease	0.021644	0.200781
5330	Allograft rejection	0.021644	0.200781
5322	Systemic lupus erythematosus	0.021799	0.200781
260	Glycine, serine and threonine metabolism	0.030951	0.2585

Abbreviations: GO—Gene Ontology; adjp—adjusted *p*-value.

Table S3. The top 20 overrepresented GO terms for DEPs in progVSCC versus d-fVSCC samples.

Name	Term	*p*-value	adjp
GO:1902578	single-organism localization	4.14×10^{-6}	0.001234
GO:0002252	immune effector process	0.000303	0.0411
GO:0009605	response to external stimulus	0.00022	0.0327
GO:0002684	positive regulation of immune system process	0.000326	0.0413
GO:0002253	activation of immune response	0.000119	0.0216
GO:0050878	regulation of body fluid levels	2.67×10^{-10}	5.03×10^{-7}
GO:0009611	response to wounding	0.000212	0.0324
GO:0002460	adaptive immune response based on somatic recombination of immune receptors built from immunoglobulin superfamily domains	7.43×10^{-7}	0.000301
GO:0002449	lymphocyte mediated immunity	4.53×10^{-7}	0.000197
GO:0002576	platelet degranulation	8.45×10^{-7}	0.000302
GO:0016485	protein processing	9.66×10^{-6}	0.00249
GO:0009914	hormone transport	0.000335	0.0413
GO:0050727	regulation of inflammatory response	3.38×10^{-5}	0.00735
GO:0006959	humoral immune response	2.16×10^{-6}	0.000679
GO:1903034	regulation of response to wounding	8.52×10^{-7}	0.000302
GO:0016064	immunoglobulin mediated immune response	3.65×10^{-10}	5.17×10^{-7}
GO:1903317	regulation of protein maturation	2.08×10^{-8}	1.31×10^{-5}
GO:0030193	regulation of blood coagulation	1.34×10^{-7}	6.34×10^{-5}
GO:0006638	neutral lipid metabolic process	0.000297	0.0411
GO:0002526	acute inflammatory response	2.06×10^{-8}	1.31×10^{-5}

Abbreviations: GO—Gene Ontology; adjp—adjusted *p*-value.

Table S4. The top 20 KEGG pathways for DEPs overrepresented in progVSCC versus d-fVSCC samples.

Name	Path	*p*-value	Adjp
4610	Complement and coagulation cascades	4.97×10^{-19}	8.9×10^{-17}
5150	Staphylococcus aureus infection	5.67×10^{-6}	0.000388
5020	Prion diseases	6.5×10^{-6}	0.000388
5322	Systemic lupus erythematosus	5.33×10^{-5}	0.00238
3320	PPAR signaling pathway	0.01385	0.496
4973	Carbohydrate digestion and absorption	0.0258	0.771
5142	Chagas disease (American trypanosomiasis)	0.0415	0.895
750	Vitamin B6 metabolism	0.0527	0.895
512	Mucin type O-Glycan biosynthesis	0.0527	0.895
5146	Amoebiasis	0.0574	0.895
4080	Neuroactive ligand-receptor interaction	0.0707	0.895
4960	Aldosterone-regulated sodium reabsorption	0.0708	0.895
4660	T cell receptor signaling pathway	0.0800	0.895
4640	Hematopoietic cell lineage	0.0837	0.895
4614	Renin-angiotensin system	0.0902	0.895
4670	Leukocyte transendothelial migration	0.0928	0.895
4920	Adipocytokine signaling pathway	0.0986	0.895
4916	Melanogenesis	0.0989	0.895
524	Butirosin and neomycin biosynthesis	0.1005	0.895
4360	Axon guidance	0.1066	0.895

Abbreviations: GO—Gene Ontology; adjp—adjusted *p*-value.

Table S5. Candidate VSCC biomarkers examined in PRM analysis.

	**Protein**
1	A2M
2	A2ML1
3	ABCC10
4	ABI3BP
5	ACTN1
6	AGR2
7	ANO1
8	CAMSAP3
9	CSTA
10	EDEM2
11	FBLN5
12	FHL1
13	GATM
14	HMGA2
15	IDO1
16	IFIT3
17	IGFBP6
18	IL1F10
19	ISG15
20	KLK14
21	KRT8
22	KRT16
23	KRT18
24	KRTDAP
25	LOR
26	MKI67
27	MX1
28	NRD1
29	OGN
30	PADI2
31	PAPLN
32	PDLIM3
33	PGLS
34	PLIN1
35	PRELP
36	PRTN3
37	PSMA5
38	PTPMT1
39	PTX3
40	RUNX1
41	S100A12
42	SFN
43	SH3BGR
44	SPRR3
45	STAT1
46	TIMP2
47	TPM3
48	UBE2C
49	WARS

Publisher's Note: MDPI stays neutral with regard to jurisdictional claims in published maps and institutional affiliations.

© 2020 by the authors. Licensee MDPI, Basel, Switzerland. This article is an open access article distributed under the terms and conditions of the Creative Commons Attribution (CC BY) license (http://creativecommons.org/licenses/by/4.0/).
